# Safety data for the use of nasal human menopausal gonadotropins: a potential novel approach for fertility treatment

**DOI:** 10.5935/1518-0557.20180078

**Published:** 2019

**Authors:** John Zhang, Zaher Merhi

**Affiliations:** 1 Reproductive Endocrinology and Infertility, New Hope Fertility Center, New York, NY; 2 Department of Obstetrics and Gynecology, Division of Reproductive Biology, New York University School of Medicine, New York, NY

**Keywords:** gonadotropin, nasal, *in vitro* fertilization, infertility

## Abstract

Some of the common side effects of the injectable gonadotropins, used during fertility treatments, are pain at the injection site, skin erythema, muscle pain, and rarely vasovagal reflex. These side effects cause inconvenience and lower patient's tolerance for fertility treatments.The purpose of this study was to evaluate the safety and efficacy of an FDA-approved dose of nasal human menopausal gonadotropins (Menopur) in women undergoing fertility treatment. Healthy regularly cycling reproductive-aged women (n=4) with infertility were enrolled. A total of 75 IU of each Menopur bottle was dissolved and placed in a nasal pump spray device (concentration of 3.75 IU/spray). Each participant was allowed to inhale a total of 2 sprays daily after which ovarian response during the follicular phase was monitored by transvaginal ultrasound and serum hormone measurement. None of the participants reported any side effects at the nasal site of drug administration. No known common side effects of the Menopur drug were reported by any of the participants. Despite adequate absorption of the nasal Menopur, as confirmed by elevated serum FSH levels while taking the nasal medication, 3 out of 4 participants did not show any follicular growth until cycle day 13 while only one participant who agreed to continue taking the medication until cycle day 20 developed one dominant follicle and had elevated serum estradiol levels. This FDA approved case series suggest that nasal route of Menopur administration seems to be safe at a very low doses and it constitutes a potential novel approach for ovarian stimulation.

## INTRODUCTION

The number of in vitro fertilization (IVF) treatment cycles and live born deliveries has continued to rise and it has been suggested that approximately 1.5% of all children born in the United States are the result of assisted reproductive technology (ART), totaling 1.1 million children since 2006 (Toner *et al.*, 2016). Gonadotropins are used during fertility treatments such as intrauterine insemination or IVF (Kamath *et al.*, 2017). Some of the common side effects of gonadotropins are pain at the injection site, skin erythema, muscle pain, and rarely fainting due to vasovagal reflex (Kinoshita *et al.*, 1999; Li & Hindle, 1993; Redfearn *et al.*, 1995). These side effects cause a lot of inconvenience and could cause a lower patient tolerance for fertility treatments. There has been great recent interest in using oral agents such as clomid and letrozole in IVF treatments (Haas & Casper, 2017; Kamath *et al.*, 2017) as well as nasal agents such as GnRH agonists (Haas & Casper, 2017). These routes will clearly decrease the need for having daily painful injections. The purpose of this study was to evaluate an alternative approach for the injectable gonadotropins in a small series of participants. Thus, we aimed to study the safety and efficacy of a very low dose of nasal human menopausal gonadotropins (Menopur, Ferring Pharmaceuticals) in women undergoing fertility treatment following an approval from the FDA.

## MATERIALS AND METHODS

### Subjects

This is a case series that included healthy regularly cycling infertile premenopausal women (n=4) who attended a private fertility clinic, aged between 18 and 45, with body mass index ranging from 19-35 kg/m^2^. Exclusion criteria included any medical condition that interferes with the health of the participant such as uncontrolled diabetes, uncontrolled hypertension, cardiac disease, or renal disease; any type of malignancy; or any mental problems that could interfere with the patient's ability to take the medication nasally. 

### Nasal Menopur administration

Due to the unusual route of administration of Menopur and in order to ensure safety of the participants (Jarow *et al.*, 2017), Investigational New Drug (IND) approval from the FDA was obtained (IND# 128484). Institutional Review Board (IRB) approval was obtained before the study started (BRANY IRB File # 15-02-467-359) and informed consent was obtained from each participant. Ovarian stimulation started on the 3^rd^ day of the menstrual cycle. A dose of 75 IU of each Menopur bottle was dissolved in 1 ml saline, and then placed in a nasal pump spray device by a New York State-certified pharmacist. Each nasal pump contained approximately 20 sprays, which makes a concentration of 3.75 IU in each spray. According to the FDA recommendation, each participant was allowed to inhale Menopur once daily (total of 2 sprays = 7.5 IU) in each nostril fearing the risk of ovarian hyperstimulation syndrome. Ovarian response was monitored during the follicular phase of the menstrual cycle by transvaginal ultrasound and standard serum hormone measurement for estradiol, progesterone, LH, and FSH. Menopur intake increases serum FSH levels, thus serum FSH levels were measured at every visit during the treatment period to ensure absorption of the medication. Compliance was assured by calling each patient every other day to confirm medication intake.

## RESULTS

The demographics and clinical characteristics of the participants are summarized in Table 1. All the participants had a regular menstrual cycle length (28-30 days) before recruitment. During participation, none of the participants reported any side effects at the nasal site of drug administration such as burning, stinging, sneezing, dryness, irritation, or congestion. No known common side effects of the Menopur drug such as headache, drowsiness, nausea, bloating, abdominal pain, or breast tenderness were reported by any of the participants. Three out of 4 participants (participant #1, 2, and 4 in Table 1), continued taking the nasal Menopur until cycle day 13 then stopped; and one participant (participant #3) continued taking the medication until cycle day 20. The last participant was the only one who developed a dominant follicle measuring 17 mm by ultrasound and had an elevated estradiol level of 183 pg/mL (Table 1). All the participants had elevated serum FSH levels at every visit during the nasal Menopur intake (up to 35 IU/mL) indicating adequate absorption of the medication.

**Table 1:** Demographics, clinical characteristics, and response to nasal Menopur administration.

Participant number	Age (years)	Body mass index (kg/m^2^)	Antral follicle count	Day 3 FSH (IU/mL)	Days of nasal Menopur intake	Peak FSH Level (IU/mL)	Peak estradiol level (pg/mL)	Size of largest follicle (mm)
1	41	27.7	7	13	9	37.7	49	6.5 (on cycle day 13)
2	34	29.2	17	6.0	8	10.0	94	10.5 (on cycle day 13)
3	40	34.2	6	9.7	16	29.0	183	17 (on cycle day 20)
4	35	26	12	3.4	8	15.7	90	10 (on cycle day 13)

## DISCUSSION 

This pilot study indicated that a total nasal dose of 7.5 IU of Menopur daily was safe but not enough to cause ovarian stimulation. Interestingly, 3 out of 4 participants (participant #1, 2, and 4 in Table 1), who had regular menstrual cycle naturally, did not develop a leading follicle in the follicular phase while taking the nasal Menopur. In one participant (participant #3 in Table 1), nasal Menopur delayed significantly the development of a leading dominant follicle. The elevated serum FSH levels few days after the start of the nasal Menopur intake indicates that nasal Menopur was systemically absorbed by the body. It is still unknown why nasal Menopur delayed or suppressed ovarian follicular development.

Nasal spray drug products contain therapeutically active ingredients (drug substances) dissolved or suspended in solutions or mixtures of excipients (e.g., buffering agents) in nonpressurized dispensers that deliver a spray containing a metered dose of the active ingredient. Injectable gonadotropin administration causes significant discomfort to patients. This new approach of nasal Menopur administration provides a practical advantage of being patient-friendly since a nasal spray provides a more convenient route of administration. This case series demonstrated that Menopur seems to be safe when administered nasally. We acknowledge the limitation of small sample size in our study and larger studies using higher doses of nasal Menopur are needed in order to achieve an adequate dose for ovarian stimulation while preventing the occurrence of any ovarian hyperstimulation syndrome cases.

In summary, we present a potential novel approach of ovarian stimulation. If the results are corroborated in a larger series of patients using higher doses, this protocol could lead to a paradigm shift in ART treatments and the inconvenient injection administration of gonadotropins in IVF may become something in the past.
